# Prevalence and Description of Hyponatremia in a Swiss Tertiary Care Hospital: An Observational Retrospective Study

**DOI:** 10.3389/fmed.2020.00512

**Published:** 2020-09-11

**Authors:** Henri Lu, Peter Vollenweider, Sébastien Kissling, Pedro Marques-Vidal

**Affiliations:** ^1^Department of Cardiology, Lausanne University Hospital and University of Lausanne, Lausanne, Switzerland; ^2^Department of Internal Medicine, Lausanne University Hospital and University of Lausanne, Lausanne, Switzerland; ^3^Service of Nephrology, Department of Internal Medicine, Lausanne University Hospital and University of Lausanne, Lausanne, Switzerland

**Keywords:** hyponatremia, epidemiology, mortality, hospital costs, risk factors

## Abstract

**Background:** Hyponatremia (serum sodium concentration <135 mEq/L) is the most common electrolyte abnormality among hospitalized patients. Our aim was to study the epidemiology of hyponatremia in hospitalized patients, as well as the short-term mortality rates, the length of stay (LOS), and associated hospital costs.

**Methods:** This retrospective cohort study included 6,539 hospitalizations in the internal medicine ward of a Swiss tertiary-care teaching hospital between January 1, 2012, and December 31, 2018 (42.7% women, mean age 69 years). Using serum sodium concentration, we identified hospitalizations with hyponatremia and calculated the prevalence of overall hyponatremia, admission hyponatremia (AH), hospital-acquired hyponatremia (HAH), and persistent hyponatremia (PH) at discharge. We also studied the impact of hyponatremia on 30-day readmissions, in-hospital and 30-day mortality, and hospital LOS and costs, using multivariable logistic regression and Cox proportional hazards models, with normal natremia as reference.

**Results:** Prevalence of overall hyponatremia was 32.5% [95% confidence interval (CI), 31.3–33.6%], while prevalence of PH among hospitalizations with AH and HAH was 33.7% (31.7–35.8%). After multivariable adjustment, hyponatremia was associated with increased hospital costs (CHF 19,025 ± 485 vs. 14,962 ± 341, *p* < 0.001) and LOS (13.4 ± 0.2 vs. 10.7 ± 0.2 days, *p* < 0.001). Increased severity of hyponatremia was associated with higher hospital costs and LOS (*p* for trend <0.001). There was a trend toward more frequent 30-day readmissions associated with hyponatremia [adjusted odds ratio (OR), 1.15 (1.01–1.31), *p* = 0.032], mainly with PH: adjusted OR = 1.41 (1.17–1.71), *p* < 0.001. No association was found between severity of hyponatremia and readmissions. Hyponatremia was associated with an increase of in-hospital [adjusted OR = 1.94 (1.49–2.53), *p* < 0.001] and 30-day mortality: adjusted OR = 1.80 (1.44–2.24), *p* < 0.001. Increased severity of hyponatremia was associated with higher in-hospital and 30-day mortality (*p* for trend < 0.001).

**Conclusions:** Hyponatremia is highly prevalent among hospitalized patients and associated with an increase of LOS, early hospital readmission, in-hospital and 30-day mortality, and hospital costs. PH was associated with a substantial increase of the risk of early hospital readmission and 30-day mortality.

## Background

Hyponatremia, usually defined as a serum sodium concentration <135 mEq/L, is the most prevalent electrolyte abnormality in hospitalized patients (1). The reported frequency of hyponatremia varies according to the health-care setting, the clinical circumstances, and the definition of hyponatremia used but can affect up to 30% of patients in some series (1–3). In a hospital setting, conditions that are associated with hyponatremia include the syndrome of inappropriate antidiuretic hormone secretion (SIADH) (4), congestive heart failure, cirrhosis, chronic kidney disease, and use of specific medications, such as thiazide diuretics (5, 6). Age is strongly associated with hyponatremia, with a relative risk ranging from 1.54 for patients aged between 41 and 50, to 5.8 for patients aged ≥81 (7). Given the increasing age of patients hospitalized in internal medicine wards in Western countries (8), the prevalence of hyponatremia is expected to increase. Hyponatremia is also associated with higher mortality: in a large multicentric study, Zilberberg and colleagues found a 55% increase in the risk of death in patients admitted with hyponatremia compared with patients with normal natremia (9). Hyponatremia is also associated with increased morbidity such as falls and fractures (5), cognitive impairment and impaired quality of life (10), increased length of stay (LOS) (11), hospitalization costs, and use of medical resources (12).

Among hospitalized patients, hyponatremia can be either present on admission or acquired during hospital stay. Up to two thirds of hyponatremia cases may be hospital-acquired (2). In Singapore, Hawkins and colleagues analyzed a large database of over 120,000 patients and showed that the prevalence of hyponatremia among hospitalized patients was 42%, with 28% at admission and 14% hospital-acquired (7). Hoorn and colleagues reported that the occurrence of severe hyponatremia during hospital stay was associated with inadequate management and treatment-related factors such as thiazide diuretics, drugs stimulating the secretion of antidiuretic hormone, hypotonic intravenous fluids, and surgery (13).

Treatment of hyponatremia remains suboptimal despite recommendations regarding its diagnosis and management. In a US hospital, Donzé and colleagues reported that 42% of 4,195 patients with diagnoses of heart failure and hyponatremia on admission still had hyponatremia at discharge (14). In this particular setting, PH was associated with an increased rate of readmission and 30-day mortality [odds ratio (OR) 1.45, 95% confidence interval (CI): 1.27–1.67].

Apart from the previous study, few have examined the consequences of persistent hyponatremia (PH) at discharge (regardless of its etiology), namely, the risk of early hospital readmission. Furthermore, few data on hyponatremia among hospitalized patients exist in Switzerland. Hence, we performed an observational, monocentric, retrospective study to investigate the epidemiology of hyponatremia (including the incidence of hospital-acquired and PH at discharge) among hospitalized patients.

## Materials and Methods

### Study Design and Patient Population

The study was designed as a retrospective cohort study and conducted in the internal medicine service of Lausanne University hospital (*Centre hospitalier universitaire vaudois* or CHUV), a large tertiary referral center and teaching hospital in Lausanne, Switzerland. This internal medicine service has 140 beds and receives over 4,000 admissions per year.

All patients aged ≥18 who stayed ≥24 h in the internal medicine wards from January 1, 2012, to December 31, 2018, were screened. For patients to be included, two conditions were necessary: (1) to have signed the general consent for research (*Consentement général pour la recherche* or CGR) form and (2) to be transferred from the emergency department or the intensive care unit (ICU). The CGR is required by the Swiss legislation on research involving humans (*Loi relative à la recherche sur l'être humain*) to use patients' clinical and biological data for research purposes. It complies with the ethical principles mentioned in the Oviedo Convention, the Declaration of Helsinki, and the Declaration of Taipei. The second inclusion criterion was added because we did not have authorization from the local Ethics Committee to include patients from other departments (e.g., surgery departments). Moreover, in our institution, the emergency department and the ICU account for the majority of patients hospitalized in the internal medicine ward. Our study was approved by the Cantonal Ethics Committee on research involving humans (CER-VD, decision dated September 3, 2019).

Patients were excluded if (1) no serum sodium concentration value was available in the first 24 h following admission; (2) they were transferred from departments or wards other than the emergency department or the ICU of the CHUV; or (3) they were transferred to another ward, department, or hospital (other than a rehabilitation unit) before the end of acute care.

The following data were retrieved from patients' electronic medical records: age; gender; main diagnosis on discharge; number of comorbidities; natremia on admission, during hospitalization, and at discharge; LOS; status at discharge (alive/dead); and any 30-day readmission in any department of the CHUV. Vital status at 30 days after discharge was retrieved from the Swiss Federal Statistical Office data.

Hospitalization costs were extracted from the CHUV financial database. The diagnosis-related group (DRG) system was also used to evaluate hospitalization costs. The DRG system has been in use in Switzerland since 2012. It classifies hospitalizations into three categories on the basis of their LOS: “inlier” (LOS is similar to nationwide average LOS for a given DRG), “highlier” (LOS is longer than nationwide average LOS), and “lowlier” (LOS is shorter than nationwide average LOS). Given the small percentage of “lowliers” (<4%), they were included in the “inlier” group.

The main diagnoses were identified by extracting the International Classification of Diseases, 10th revision (ICD-10), codes in discharge letters, and were categorized into the following groups: infectious diseases, cancers, pulmonary diseases, cardiac diseases, liver diseases, neurological diseases, endocrine disorders, psychiatric disorders, and others (Supplementary Table 1). Kidney diseases represented <0.5% of main diagnoses and were included in the “other diagnoses” category.

All data were extracted and anonymized by the Data Science & Research (DS&R) group, which is the only entity authorized to extract data from the CHUV databases. After extraction, the source database provided by the DS&R group was converted into a statistical-friendly format. All modifications and analyses were performed via programming, a new database was created, and no changes were being made to the original database. All files were stored in a secured server within the research folder of the internal medicine ward. Access to this folder was limited to the principal investigator and the persons in charge of the analyses.

### Endpoints

The main endpoint was the prevalence of overall hyponatremia, admission hyponatremia (AH), hospital-acquired hyponatremia (HAH), and PH.

Hyponatremia was defined as a serum sodium concentration <135 mEq/L and further categorized into mild (130–135 mEq/L), moderate (125–129 mEq/L), and severe (<125 mEq/L) (6). Hyponatremia at admission was defined by hyponatremia evidenced during the first 24 h of hospitalization. HAH was defined as hyponatremia diagnosed after the first 24 h of hospitalization, with natremia within normal range (135–145 mEq/L) on admission. Overall hyponatremia was defined as AH or HAH. Lastly, PH was defined as hyponatremia evidenced at admission or after the first 24 h of hospitalization, and on the last available blood test before discharge.

Secondary objectives included studying (1) the demographic and clinical characteristics (age, gender, and main diagnosis during hospitalization) associated with hyponatremia, with normal natremia as reference; (2) the impact of hyponatremia on hospital LOS and costs, 30-day readmissions, in-hospital all-cause mortality, and all-cause 30-day mortality, using normal natremia as reference.

### Covariates

To account for potential confounders when comparing hospital LOS and costs, readmissions, and all-cause mortality rates between patients with and without hyponatremia, the following covariates were selected on the basis of previous reports (14–16): age group (four categories: <60, 60–69, 70–79, and >80 years old), gender, main diagnosis group (eight categories, as mentioned above), and the number of comorbidities.

### Statistical Analyses

Statistical analyses were conducted using Stata version 16.0 for Windows^®^ (Stata Corp, College Station, TX, USA). As some patients were hospitalized several times, the unit of analysis was the total number of hospitalizations (and not the total number of patients), unless specified otherwise. Results were expressed as number and (percentage) for categorical variables and as average ± standard deviation (SD) or as median [interquartile range (IQR)] for continuous variables. Between-group bivariate comparisons were performed using chi-square for categorical variables and Student's *t*-test, analysis of variance (ANOVA), or Kruskal–Wallis test for continuous variables. As LOS and costs showed a right-skewed distribution, a multivariable analysis was performed on log-transformed values. Multivariable analyses were conducted using multilevel mixed-effects linear regression for continuous variables and multilevel mixed-effects logistic regression for categorical variables to take into account multiple hospitalizations for the same patient, or plain ANOVA and logistic regression when single individual data were used. Results were expressed as multivariable-adjusted mean ± standard error for continuous variables and as multivariable-adjusted OR and (95% CI) for categorical variables. For mortality analyses, only the last hospitalization of each patient was considered. Mortality was assessed using Cox regression, and results were expressed as hazard ratios (HRs) and (95% CI) (17). Multivariable analyses were performed adjusting for age (four categories), gender, main cause for hospitalization (eight categories), and number of associated comorbidities. *Post-hoc* trend analyses were performed using the contrast p. command of Stata.

Due to the number of tests performed and as suggested by others (18), a conservative value of 0.005 was used to define statistical significance.

## Results

From January 1, 2012, to December 31, 2018, a total of 18,241 hospitalizations in the internal medicine wards of the CHUV, with available sodium values in the first 24 h after admission, were recorded. A total of 4,307 patients, representing 7,133 hospitalizations (35% of total hospitalizations), had signed the CGR and were eligible for inclusion. A total of 594 hospitalizations were excluded because of missing data or exclusion criteria, with 6,539 hospitalizations included in the analyses. The characteristics of the included and excluded hospitalizations are summarized in Supplementary Table 2: included patients were significantly older and had significantly shorter LOS.

### Prevalence and Determinants of Hyponatremia

The trends and prevalence of overall, AH, HAH, and PH are summarized in Table 1. Prevalence of AH was 24.7% (95% CI, 23.7–25.8%), and prevalence of HAH among participants devoid of AH was 10.3% (95% CI, 9.4–11.1%). Interestingly, nearly one third of patients had PH at discharge (33.7%). Most cases of hyponatremia were mild (69.1%), with 20.2% moderate and 10.7% severe.

**Table 1:** Annual trends in the prevalence of hyponatremia: Lausanne University Hospital, 2012–2018.

	**2012**	**2013**	**2014**	**2015**	**2016**	**2017**	**2018**	**All**
All hospitalizations	376	885	963	1,014	982	1,052	1,267	6,539
**Hyponatremia, all**								
Overall	78 (20.7)	273 (30.9)	309 (32.1)	278 (27.4)	365 (37.2)	358 (34)	462 (36.5)	2,123 (32.5)
Admission	58 (15.4)	205 (23.2)	218 (22.6)	197 (19.4)	277 (28.3)	289 (27.6)	371 (29.3)	1,615 (24.7)
Hospital-acquired^a^	20 (6.3)	68 (10.0)	91 (12.2)	81 (9.9)	87 (12.4)	69 (9.1)	89 (10.0)	505 (10.3)
Persistent^b^	16 (20.5)	85 (31.1)	111 (35.9)	72 (25.9)	133 (36.4)	129 (36.0)	170 (36.8)	716 (33.7)
**Severity of hyponatremia**								
Mild (130–135 mEq/L)	52 (66.7)	173 (63.4)	214 (69.3)	192 (69.1)	258 (70.7)	242 (67.6)	336 (72.7)	1,467 (69.1)
Moderate (125–129 mEq/L)	16 (20.5)	66 (24.2)	64 (20.7)	56 (20.1)	70 (19.2)	74 (20.7)	82 (17.8)	428 (20.2)
Severe (<125 mEq/L)	10 (12.8)	34 (12.5)	31 (10.0)	30 (10.8)	37 (10.1)	42 (11.7)	44 (9.5)	228 (10.7)

*Results are expressed as number of hospitalizations (percentage)*.

a *Among hospitalizations without hyponatremia at admission*.

b *Among hospitalizations with hyponatremia at admission or hospital-acquired hyponatremia*.

The demographic and clinical characteristics of hospitalizations with and without hyponatremia are shown in Table 2. Hospitalizations with severe hyponatremia had a lower frequency of women and a lower frequency of subjects aged <60. Furthermore, the main diagnoses on discharge groups were significantly different according to the severity of hyponatremia, with a higher proportion of endocrine disorders associated with severe hyponatremia.

**Table 2:** Demographic and clinical characteristics of hospitalizations according to categories of sodium levels.

	**All**	**Normal**	**Mild**	**Moderate**	**Severe**	***p* value**
*N*	6,539	4,416	1,467	428	228	
Women (%)	2,794 (42.7)	1,925 (43.6)	564 (38.5)	187 (43.7)	118 (51.8)	<0.001
Age groups (%)						<0.001
<60	1,561 (23.9)	1,036 (23.5)	378 (25.8)	104 (24.3)	43 (18.9)	
60–69	1,340 (20.5)	817 (18.5)	343 (23.4)	120 (28.0)	60 (26.3)	
70–79	1,597 (24.4)	1,095 (24.8)	352 (24.0)	93 (21.7)	57 (25.0)	
≥80	2,041 (31.2)	1,468 (33.2)	394 (26.9)	111 (25.9)	68 (29.8)	
Main diagnosis on discharge (%)						<0.001
Pulmonary disease	1,669 (25.5)	1,228 (27.8)	333 (22.7)	79 (18.5)	29 (12.7)	
Heart disease	1,121 (17.1)	826 (18.7)	195 (13.3)	56 (13.1)	44 (19.3)	
Cancer	537 (8.2)	342 (7.7)	138 (9.4)	43 (10.1)	14 (6.1)	
Neurological disease	326 (5.0)	234 (5.3)	57 (3.9)	22 (5.1)	13 (5.7)	
Liver disease	211 (3.2)	86 (2.0)	89 (6.1)	22 (5.1)	14 (6.1)	
Endocrine disorders	139 (2.1)	42 (1.0)	46 (3.1)	19 (4.4)	32 (14.0)	
Psychiatric disorders	85 (1.3)	57 (1.3)	18 (1.2)	7 (1.6)	3 (1.3)	
Other diseases	2,451 (37.5)	1,601 (36.3)	591 (40.3)	180 (42.1)	79 (34.7)	

*Hyponatremia values include acquired-hyponatremia and hospital-acquired hyponatremia: Lausanne University Hospital, 2012–2018*.

*Results are expressed as number of hospitalizations (percentage). Between-group comparisons performed using chi-square test*.

The demographic and clinical characteristics of hospitalizations with and without PH are reported in Supplementary Table 3. Hospitalizations with PH tended to have a higher frequency of women and different main diagnosis groups, while no differences were found for age groups.

Supplementary Table 4 shows the number of sodium measurements and the natremia values, according to categories of sodium levels. The number of measurements increased with the severity of hyponatremia. Increases in natremia across hospital stay were stronger among the most severe categories. No differences were observed for PH as expressed by the median rises of natremia from admission to discharge.

### Hyponatremia, Length of Stay, Hospital Costs, and Readmissions

The associations between natremia levels and hospital costs and DRG categories are summarized in Table 3. Admissions with hyponatremia incurred higher costs and were more frequently DRG highliers than admissions with normal natremia, and this difference persisted after a multivariate analysis. The severity of hyponatremia was positively associated with an increase in hospital costs and in the likelihood of being DRG highlier. Non-PH was associated with an increase in hospital costs and the likelihood of being DRG highlier, while PH was not.

**Table 3:** Bivariate and multivariable analysis of the association between natremia levels and hospital costs or diagnosis-related group (DRG) categories: Lausanne University Hospital, 2012–2018.

	**Hospital costs (CHF)**				**Diagnosis-related group categories**				
	**Bivariate**		**Multivariable**		**Bivariate**			**Multivariable**	
	**Median [IQR]**	***p* value**	**Mean ± SE**	***p* value^§^**	**Inlier**	**Highlier**	***p* value**	**OR (95% CI)**	***p* value^§^**
Natremia levels		<0.001		<0.001			<0.001		
Normal	10,299 [7,029–16,025]		14,962 ± 341		3,851 (69.6)	565 (56.3)		1 (reference)	
Decreased	13,142 [8,644–22,586]		19,025 ± 485		1,684 (30.4)	439 (43.7)		1.66 (1.41–1.95)	<0.001
Natremia levels		<0.001		<0.001			<0.001		
Normal	10,299 [7,029–16,025]		14,920 ± 340		3,851 (69.6)	565 (56.3)		1 (reference)	
Mild (130–135 mEq/L)	12,610 [8,470–21,318]		17,516 ± 567		1,194 (21.6)	273 (27.2)		1.49 (1.24–1.78)	<0.001
Moderate (125–129 mEq/L)	14,577 [9,789–26,675]		22,210 ± 1,030		317 (5.7)	111 (11.1)		2.19 (1.66–2.88)	<0.001
Severe (<125 mEq/L)	14,628 [9,232–27,542]		23,395 ± 1,422		173 (3.1)	55 (5.5)		1.95 (1.34–2.82)	<0.001
*p* value for trend			<0.001					<0.001	
Persistent hyponatremia		<0.001		0.001			<0.001		
Normal	10,299 [7,029–16,025]		14,966 ± 341		3,851 (69.6)	565 (56.3)		1 (reference)	
Non-persistent	13,725 [9,073–24,295]		20,188 ± 582		1,084 (19.6)	323 (32.2)		1.90 (1.58–2.27)	<0.001
Persistent	12,305 [7,932–18,851]		16,695 ± 807		600 (10.8)	116 (11.6)		1.23 (0.96–1.57)	0.109
*p* value for trend^‡^			0.001					0.109	

*CHF, Swiss Francs; CI, confidence interval; IQR, interquartile range; OR, odds ratio; SE, standard error; Inlier, length of stay is similar to nationwide average for a given DRG; highlier, length of stay is longer than nationwide average*.

‡ *Multivariable model*.

§ *Based on log-transformed data*.

*Results are expressed as median [interquartile range] or multivariable-adjusted mean ± standard error for continuous variables and as number of hospitalizations (percentage) or multivariable-adjusted odds ratio (95% confidence interval) for categorical variables. Between-group bivariate analysis performed using Kruskal–Wallis test for continuous variables and chi-square for categorical variables. Multivariable analysis conducted using multilevel mixed-effects linear regression for continuous variables and multilevel mixed-effects logistic regression for categorical variables. Multivariable analyses were performed adjusting for age (four categories), gender, main cause for hospitalization (eight categories), and number of associated comorbidities*.

The associations between natremia levels and LOS and 30-day readmissions are summarized in Table 4. Admissions with hyponatremia had a longer LOS and a 15% higher likelihood of readmission. LOS increased with the severity of hyponatremia, but no association was found between severity of hyponatremia and readmission. Non-PH had a longer LOS while PH presented a higher likelihood of readmission.

**Table 4:** Bivariate and multivariable analysis of the association between natremia levels and lengths of stay or 30-day unplanned readmissions: Lausanne University Hospital, 2012–2018.

	**Length of stay (days)**				**Readmissions**				
	**Bivariate**		**Multivariable**		**Bivariate**			**Multivariable**	
	**Median [IQR]**	***p* value**	**Mean ± SE**	***p* value^§^**	**No**	**Yes**	***p* value**	**OR (95% CI)**	***p* value**
Natremia levels		<0.001		<0.001			<0.001		
Normal	8 [5–13]		10.7 ± 0.2		2,946 (69.4)	1,470 (64.1)		1 (reference)	
Decreased	10 [7–17]		13.4 ± 0.2		1,301 (30.6)	822 (35.9)		1.15 (1.01–1.31)	0.031
Natremia levels		<0.001		<0.001			<0.001		
Normal	8 [5–13]		10.6 ± 0.2		2,946 (69.4)	1,470 (64.1)		1 (reference)	
Mild (130–135 mEq/L)	10 [7–16]		12.6 ± 0.3		910 (21.4)	557 (24.3)		1.14 (0.98–1.31)	0.080
Moderate (125–129 mEq/L)	12 [8–20]		15.2 ± 0.5		253 (6.0)	175 (7.6)		1.22 (0.96–1.54)	0.106
Severe (<125 mEq/L)	12 [8–21]		16.1 ± 0.7		138 (3.3)	90 (3.9)		1.14 (0.82–1.56)	0.438
*p* value for trend			<0.001					0.381	
Persistent hyponatremia		<0.001		0.003			<0.001		
Normal	8 [5–13]		10.7 ± 0.2		2,946 (69.4)	1,470 (64.1)		1 (reference)	
Non-persistent	11 [7–19]		14.2 ± 0.3		890 (21)	517 (22.6)		1.04 (0.89–1.20)	0.644
Persistent	10 [6–15]		11.9 ± 0.4		411 (9.7)	305 (13.3)		1.41 (1.17–1.71)	<0.001
*p* value for trend^‡^			0.003					<0.001	

*CI, confidence interval; IQR, interquartile range; OR, odds ratio; SE, standard error*.

§ *Multivariable model*.

‡ *Based on log-transformed data*.

*Results are expressed as median [interquartile range] or multivariable-adjusted mean ± standard error for continuous variables and as number of hospitalizations (percentage) or multivariable-adjusted odds ratio (95% confidence interval) for categorical variables. Between-group bivariate analysis performed using Kruskal–Wallis test for continuous variables and chi-square for categorical variables. Multivariable analysis conducted using multilevel mixed-effects linear regression for continuous variables and multilevel mixed-effects logistic regression for categorical variables. Multivariable analyses were performed adjusting for age (four categories), gender, main cause for hospitalization (eight categories), and number of associated comorbidities*.

### Hyponatremia and Mortality

The associations between hyponatremia and in-hospital or 30-day mortality are summarized in Table 5. Overall hyponatremia was associated with an almost doubling of in-hospital mortality, and the association increased with the severity of the condition. Similar findings were obtained for 30-day mortality. Non-PH and PH were associated an increase of in-hospital and 30-day mortality, the increase being stronger for PH.

**Table 5:** Bivariate and multivariable analysis of the association between natremia levels and in-hospital or 30-day mortality: Lausanne University Hospital, 2012–2018.

	**In-hospital mortality**					**30-day mortality**				
	**Bivariate**			**Multivariable**		**Bivariate**			**Multivariable**	
	**Alive**	**Dead**	***p* value**	**HR (95% CI)**	***p* value**	**Alive**	**Dead**	***p* value**	**HR (95% CI)**	***p* value**
Natremia levels			<0.001							
Normal	2,560 (68.2)	138 (50.2)		1 (reference)		2,473 (68.5)	225 (53.2)	<0.001	1 (reference)	
Decreased	1,196 (31.8)	137 (49.8)		1.82 (1.43–2.33)	<0.001	1,135 (31.5)	198 (46.8)		1.68 (1.37–2.04)	<0.001
Natremia levels			<0.001							
Normal	2,560 (68.2)	138 (50.2)		1 (reference)		2,473 (68.5)	225 (53.2)	<0.001	1 (reference)	
Mild (130–135 mEq/L)	825 (22)	78 (28.4)		1.48 (1.12–1.97)	0.007	792 (22.0)	111 (26.2)		1.34 (1.06–1.69)	0.013
Moderate (125–129 mEq/L)	230 (6.1)	38 (13.8)		2.54 (1.75–3.67)	<0.001	209 (5.8)	59 (14.0)		2.52 (1.88–3.39)	<0.001
Severe (<125 mEq/L)	141 (3.8)	21 (7.6)		3.00 (1.88–4.79)	<0.001	134 (3.7)	28 (6.6)		2.47 (1.65–3.69)	<0.001
*p* value for trend				<0.001					<0.001	
Persistent hyponatremia			<0.001							
Normal	2,560 (68.2)	138 (50.2)		1 (reference)		2,473 (68.5)	225 (53.2)		1 (reference)	
Non-persistent	797 (21.2)	77 (28.0)		1.57 (1.18–2.09)	0.002	767 (21.3)	107 (25.3)		1.39 (1.09–1.76)	0.007
Persistent	399 (10.6)	60 (21.8)		2.29 (1.68–3.12)	<0.001	368 (10.2)	91 (21.5)		2.20 (1.72–2.83)	<0.001
*p* value for trend^‡^				<0.001					<0.001	

*HR, hazard ratio; CI, confidence interval*.

‡ *Multivariable model*.

*Results are expressed as number (column percentage) for bivariate comparisons and as multivariable-adjusted odds ratio and (95% confidence interval) for multivariable comparisons. Between-group comparisons performed using chi-square (bivariate) and Cox regression (multivariable). Multivariable analyses were performed adjusting for age (four categories), gender, main cause for hospitalization (eight categories), and number of associated comorbidities*.

## Discussion

The main results of our study are (1) hyponatremia was very common in hospitalized patients, one patient out of four having AH, one patient out of 10 developing HAH, and, among patients with AH or HAH, one out of three (33.7%) having PH; (2) hyponatremia was associated with increased readmissions, LOS, hospital costs, in-hospital, and 30-day mortality.

### Prevalence and Determinants of Hyponatremia

The overall prevalence of hyponatremia in our study was close to 33%, a value almost 10-fold higher than reported in a previous retrospective study conducted in three hospitals in Basel, Switzerland (368/10,500 admissions or 3.5%) (19). A possible explanation is that the authors used diagnostic codes in discharge letters to identify hyponatremia; this might have led to an underestimation, as hyponatremia is usually underreported in hospital codifications (20). Conversely, our estimation is in the same range as previous reports: Hawkins using data from over 120,000 patients in a Singaporean hospital, found a prevalence of hyponatremia of ~42% (7); Hoorn et al. using data from 2,900 patients in a Dutch hospital, reported an incidence of hospital-associated hyponatremia of ~30% (13). Hence, our results suggest that hyponatremia (all types) is a common condition among patients hospitalized in internal medicine.

A striking finding was the relatively high rate of HAH and PH. In a previous single-center study including 53,236 hospitalizations, Wald et al. found an even higher incidence of HAH (38.2%) (15), which may be partly explained by the use of different cutoff values: hyponatremia was defined as serum sodium concentration <138 mEq/L. Concerning PH, Donzé et al. found an incidence of 41.9% among 4,295 patients hospitalized with a diagnosis of congestive heart failure (14), while Greenberg and colleagues, in a large multicentric American and European prospective study, reported that as many as 78% of patients, initially hospitalized with hypervolemic or euvolemic hyponatremia, still had PH at discharge (21). The mechanisms underlying HAH and PH are probably different, as HAH may be the marker of a transient event during hospitalization while PH is possibly the marker of a chronic disease. The high prevalence of HAH may be due to several factors such as a lack of medical awareness, inaccurate diagnostic, and volume status assessment (21, 22), and lack of a hospital warning system (13), while PH may be partly due to variable effectiveness of available treatments (6) or the fact that hyponatremia is frequently considered as the consequence of an underlying disorder (which diverts attention from hyponatremia itself).

### Hyponatremia, Lengths of Stay, Hospital Costs, and Readmissions

Hyponatremia was associated with increased LOS and hospitalization costs, a finding in agreement with the literature (1, 9, 15). In a large meta-analysis including almost 4,000,000 patients from the United States and Europe, Corona et al. reported that hyponatremia was associated with an increase of LOS (+3.30 days, 95% CI, 2.90–3.71) and of hospital costs up to $3,000 compared with normonatremia (23). Our study extended these data by adding there was a “dose–response” relationship between LOS or hospitalization costs, and the severity of hyponatremia. In a Swiss setting, Althaus and Krapf found a median average LOS of 9 days, which was slightly lower to ours (19). However, the study was not limited to internal medicine wards and may have included patients with fewer comorbidities.

The severity of the hyponatremia was not significantly associated with the risk of early readmission. However, hyponatremia overall was associated with an increased risk of early hospital readmission, as was PH, but not non-PH. Accordingly, the last in-hospital sodium level is part of the HOSPITAL score, which has been developed to predict 30-day potentially avoidable hospital readmissions (24). Importantly, the risk of early hospital readmission was mainly found for PH, as opposed to normalized natremia on discharge. This again highlights the fact that PH should not be overlooked before discharging patients.

### Hyponatremia and Mortality

Our in-hospital and 30-day mortality rates were in the same range as previous reports: Wald et al. reported AH and HAH (with hyponatremia defined as serum sodium concentration <138 mEq/L) to be associated with an increase of in-hospital mortality [respectively adjusted OR of 1.52 (95% CI, 1.36–1.69) and adjusted OR of 1.66 (95% CI, 1.39–1.98)] (15), while Holland-Bill et al. in a large Danish study including 41,803 hospitalized patients, found hyponatremia to be associated with an increased 30-day mortality risk [adjusted relative risk, 1.5 (95% CI, 1.4–1.5)] (25). In our study, in-hospital mortality was significantly increased, even in cases of mild hyponatremia, while PH was associated with a substantial increase of 30-day mortality. Compared with AH, HAH was associated with a 66% increase in the adjusted odds of in-hospital mortality. Whether hyponatremia by itself contributes to mortality or reflects the severity of the underlying diseases remains unclear. We also observed a “dose–response” relationship between in-hospital or 30-day mortality risk, and hyponatremia severity, in contrast with what was previously published (25).

### Implications for Clinical Practice

Whether hyponatremia should solely be considered as a simple consequence of another disease or as a main issue in itself remains to this day debatable (26). Further studies are needed to establish if rapid diagnosis, close clinical, and biological monitoring, and adequate management of hyponatremia could reduce short-term mortality risk, LOS and associated costs, and early readmission and improve quality of life. Accurate clinical volume status (22) and the use of specific and complete testing [for example, urine and plasma osmolality, as well as urine sodium at a minimum for a proper diagnosis of SIADH (6)] are paramount. Moreover, outpatient follow-up should not be overlooked, as close follow-up visits may help to prevent relapse and development of chronic hyponatremia (19).

### Strengths and Limitations

This study has several strengths. Firstly, it is based on a cohort of patients with a wide array of comorbid conditions, from an acute care setting, who had complete available follow-up data, *via* hospital and government registries. The size of the cohort allowed us to explore multiple outcomes related to hyponatremia while adjusting for variable potential confounders. We were able to investigate less studied outcomes, such as the proportion of PH and the risk of early readmission, in addition to studying common endpoints usually described in hyponatremia (prevalence, risk of mortality, and LOS). Secondly, contrary to several previous studies, which used diagnostic codes in discharge letters to identify patients with hyponatremia, we were able to directly use plasma sodium levels in our analyses. Using diagnostic codes, only, may lead to an underestimation, as hyponatremia is usually underreported in hospital codifications (20).

This study has several limitations. Firstly, the study was conducted in a single center in a high-volume Swiss tertiary hospital and may not be applicable to other settings. Still, the characteristics of the patients are similar to those found in general internal medicine wards. Secondly, almost two thirds of patients eligible were excluded because they did not sign the CGR. This might have created a selection bias, as younger people tend to provide consent more frequently (27); still, the direction and magnitude of the bias are hard to estimate. Thirdly, our epidemiological study was limited in the identification of important clinical and biological determinants of hyponatremia (volume status, plasma and urine electrolytes, and osmolality), which are critical to understand its diagnostic, therapeutic, and prognosis significations. Also, a causal relation between hyponatremia and the main diagnosis and/or underlying medical conditions cannot be concluded. Finally, we did not assess medical therapy on admission and during hospital stay. Indeed, medical therapy on admission is not systematically documented in the software used in our institution. Moreover, when medical therapy on admission is documented, it may not be reliable. Since therapy on admission was unavailable, we chose not to include therapy received during hospital stay either, because analyzing the association between hospital treatment only and all categories of hyponatremia (AH, HAH, and PH) would be challenging. Therefore, we were unable to analyze if some of the cases of hyponatremia were iatrogenic or if inadequate management was partly responsible for PH. Knowing the causative diagnosis and the determinant factors for PH as well as knowing the reasons for readmission and 30-day mortality would be critical to improve our understanding and the treatment of these patients.

## Conclusions

Hyponatremia remains highly prevalent among hospitalized patients and associated with an increase of early hospital readmission, in-hospital and 30-day mortality, LOS, and hospital costs. PH was associated with a substantial increase of the risk of early hospital readmission and 30-day mortality.

## Data Availability Statement

Due to lack of consent for posting, individual data cannot be made publicly available to other investigators as it would not comply with the Swiss legislation [Federal Act on Research involving Human Beings (Human Research Act, HRA), of 30 September 2011 (Status as of 1 January 2020)]. For more details: www.admin.ch/opc/en/classified-compilation/20061313/index.html. Requests to access the datasets should be directed to henri.lu@chuv.ch.

## Ethics Statement

The protocol of this study was reviewed and approved by the Cantonal Ethics Committee on research involving humans (CER-VD, decision dated September 3rd, 2019). The participants provided their written informed consent to participate in this study. Written informed consent was obtained from the individuals for the publication of any potentially identifiable images or data included in this article.

## Author Contributions

HL designed the study, interpreted the data, and wrote most of the manuscript. PM-V analyzed the data and wrote part of the manuscript. PV and SK revised the manuscript for important intellectual content. All authors read and approved the final manuscript.

## Conflict of Interest

The authors declare that the research was conducted in the absence of any commercial or financial relationships that could be construed as a potential conflict of interest.
