# Supplementary material for: Prevalence and Description of Hyponatremia in a Swiss Tertiary Care Hospital: An Observational Retrospective Study
Source: Front Med (Lausanne). 2020 Sep 11;7:512. doi: 10.3389/fmed.2020.00512 (PMC7517335; doi:10.3389/fmed.2020.00512)
Supplement: Supplementary file 1 [file Table_1.docx]

**Prevalence and description of hyponatremia in a Swiss tertiary care hospital: an observational retrospective study**

Henri Lu^1^, Peter Vollenweider^2^, Sébastien Kissling^3^, Pedro Marques-Vidal^2^

^1^: Department of cardiology, Lausanne University Hospital and University of Lausanne, Lausanne, Switzerland

^2^: Department of internal medicine, Lausanne University Hospital and University of Lausanne, Lausanne, Switzerland

^3^: Service of nephrology, Department of internal medicine, Lausanne University Hospital and University of Lausanne, Lausanne, Switzerland

**Supplementary table 1**. International classification of diseases – 10^th^ edition (ICD-10) codes used to define the main causes for admission.

|  | **ICD-10 codes** |
| --- | --- |
| Infectious diseases | A00-B99 |
| Cancers | C00-D49, R97 |
| Pulmonary diseases | J00-J99, Q30-Q34, R04-R09, R91 |
| Heart diseases | I05-I52, Q20-Q28, R00-R03 |
| Liver diseases | K70-K77, R16-R18 |
| Neurological diseases | G00-G99, F01-F09, I60-I69, Q00-Q07, R25-R29, R40-R44, R51, R56, R90 |
| Endocrine disorders | E00-E36 |
| Psychiatric disorders | F10-F69, F90-F99, R45, R46 |
| Other diseases | All other codes |

**Supplementary table 2**. Comparison between included and excluded admissions. Lausanne University Hospital, 2012-2018.

|  | **Included (N=6539)** | **Excluded (N=594)** | **P-value** |
| --- | --- | --- | --- |
| Women (%) | 2794 (42.7) | 246 (41.4) | 0.535 |
| Age (years) | 69.2 ± 16.3 | 65.6 ± 16.9 | <0.001 |
| Length of stay (days) | 9 [6 - 14] | 13 [8 - 23] | <0.001 § |

Results are expressed as number of admissions (column percentage) for categorical variables and as average ± standard deviation or median [interquartile range] for continuous variables. Between-group comparisons performed using chi-square for categorical variables and student’s t-test or Kruskal-Wallis test (§) for continuous variables.

**Supplementary table 3.** Demographic and clinical characteristics of hospital stays according to persistent hyponatremia. Lausanne University Hospital, 2012-2018.

|  | **No** | **Yes** | **P-value** |
| --- | --- | --- | --- |
| N | 1407 | 716 |  |
| Women (%) | 602 (42.8) | 267 (37.3) | 0.015 |
| Age groups (%) |  |  | 0.453 |
| < 60 | 359 (25.5) | 166 (23.2) |  |
| 60-69 | 334 (23.7) | 189 (26.4) |  |
| 70-79 | 337 (24.0) | 165 (23.0) |  |
| ≥ 80 | 377 (26.8) | 196 (27.4) |  |
| Main diagnosis on discharge |  |  | <0.001 |
| Cancer | 108 (7.7) | 87 (12.2) |  |
| Pulmonary disease | 317 (22.5) | 124 (17.3) |  |
| Heart disease | 184 (13.1) | 111 (15.5) |  |
| Liver disease | 68 (4.8) | 57 (8.0) |  |
| Neurological disease | 61 (4.3) | 31 (4.3) |  |
| Endocrine disorders | 70 (5.0) | 27 (3.8) |  |
| Psychiatric disorders | 17 (1.2) | 11 (1.5) |  |
| Other diseases | 582 (41.4) | 268 (37.4) |  |

Results are expressed as number of hospitalizations (percentage). Between-group comparisons performed using chi-square.

**Supplemental table 4**. Number of value of sodium measurements according to categories of sodium levels. Lausanne University Hospital, 2012-2018.

|  | **Number of measurements** | **P-value** | **Na first measurement (mmol/L)** | **P-value** | **Na last measurement**  **(mmol/L)** | **P-value** | **Difference last-first (mmol/l)** | **P-value** |
| --- | --- | --- | --- | --- | --- | --- | --- | --- |
| Natremia levels |  | <0.001ǂ |  | NR |  | NR |  | <0.001ǂ |
| Normal | 4 [2 ; 6] |  | 140 ± 3 |  | 140 ± 3 |  | 0 [-1 ; 2] |  |
| Decreased | 6 [4 ; 10] |  | 132 ± 6 |  | 136 ± 4 |  | 4 [0 ; 7] |  |
| Natremia levels |  | <0.001ǂ |  | NR |  | <0.001 |  | <0.001ǂ |
| Normal | 4 [2 ; 6] |  | 140 ± 3 |  | 140 ± 3 |  | 0 [-1 ; 2] |  |
| Mild (130-135 mEq/L) | 5 [3 ; 8] |  | 134 ± 3 |  | 137 ± 3 |  | 3 [-1 ; 6] |  |
| Moderate (125-129 mEq/L) | 8 [5 ; 12] |  | 130 ± 4 |  | 135 ± 5 |  | 5 [1 ; 10] |  |
| Severe (<125 mq/L) | 10 [7 ; 16] |  | 124 ± 8 |  | 134 ± 6 |  | 12 [5 ; 17] |  |
| Persistent hyponatremia |  | <0.001ǂ |  | NR |  | NR |  | NR |
| Normal | 4 [2 ; 6] |  | 140 ± 3 |  | 140 ± 3 |  | 0 [-1 ; 2] |  |
| Non-persistent | 6 [4 ; 11] |  | 133 ± 5 |  | 138 ± 3 |  | 5 [2 ; 9] |  |
| Persistent | 6 [4 ; 9] |  | 132 ± 6 |  | 132 ± 3 |  | 0 [-3 ; 3] |  |

NR, not relevant. Results are expressed as median [interquartile range] or mean ± standard deviation. Between-group bivariate analysis performed using student’s or Kruskal-Wallis test
